# Setting the global health agenda: The influence of advocates and ideas on political priority for maternal and newborn survival

**DOI:** 10.1016/j.socscimed.2016.08.013

**Published:** 2016-10

**Authors:** Stephanie L. Smith, Jeremy Shiffman

**Affiliations:** a School of Public Administration, The University of New Mexico, Social Sciences Bldg. 3008, MSC05 3100, 1 University of New Mexico, Albuquerque, NM, 87131-0001, USA; b Department of Public Administration and Policy, School of Public Affairs, American University, 4400 Massachusetts Ave., NW, Washington, DC, 20016-8070, USA

**Keywords:** Global health, Policy agenda, Political priority, Networks, Framing, Maternal, Newborn

## Abstract

This study investigates a puzzle concerning global health priorities—why do comparable issues receive differential levels of attention and resources? It considers maternal and neonatal mortality, two high-burden issues that pertain to groups at risk at birth and whose lives could be saved with effective intrapartum care. Why did maternal survival gain status as a global health priority earlier and to a greater degree than newborn survival? Higher mortality and morbidity burdens among newborns and the cost-effectiveness of interventions would seem to predict that issue's earlier and higher prioritization. Yet maternal survival emerged as a priority two decades earlier and had attracted considerably more attention and resources by the close of the Millennium Development Goals era. This study uses replicative process-tracing case studies to examine the emergence and growth of political priority for these two issues, probing reasons for unexpected variance. The study finds that maternal survival's grounding as a social justice issue spurred growth of a strong and diverse advocacy network and aligned the issue with powerful international norms (e.g. expectations to advance women's rights and the Millennium Development Goals), drawing attention and resources to the issue over three decades. Newborn survival's disadvantage stems from its long status as an issue falling under the umbrellas of maternal and child survival but not fully adopted by these networks, and with limited appeal as a public health issue advanced by a small and technically focused network; network expansion and alignment with child survival norms have improved the issue's status in the past few years.

## Highlights

• Despite comparable burden maternal has higher agenda status than newborn survival.
• Diverse membership (beyond health circles) is an asset to health advocacy networks.
• These networks must frame issues to build broad political coalitions.
• International policy expectations (norms) affect global health issue attention.

**Fig. 1:**
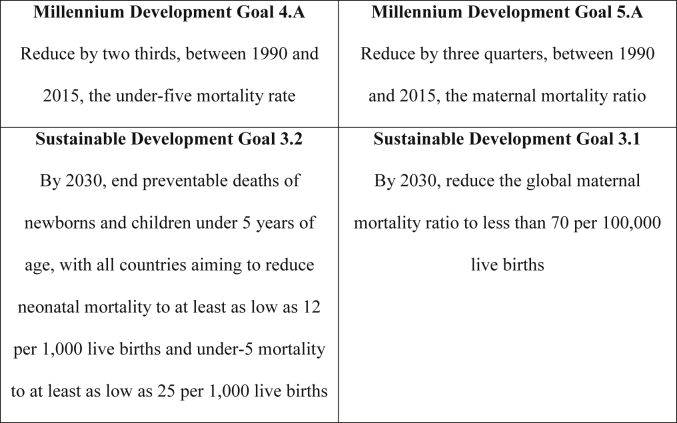
International development goals. Sources: Millennium Development Goals (http://www.un.org/millenniumgoals/); Sustainable Development Goals (http://www.un.org/sustainabledevelopment/health/).

Maternal and neonatal mortality reduction appear prominently among the freshly minted United Nations Sustainable Development Goals (SDGs; Fig. 1) for health, but the issues have not always been on the international health or development policy agendas. Many factors understood to facilitate the ascendance of issues on policy agendas apply to maternal and newborn survival, concerned respectively with reducing preventable deaths to pregnant women and newborn babies. Their global mortality and morbidity burdens are high; an estimated 2.7 million neonates and 303,000 women die annually, while neonatal conditions comprise 202 million and maternal conditions 16 million disability-adjusted life-years (DALYs) (Murray et al., 2012, UNICEF et al., 2015, World Health Organization et al., 2015). And, both issues pertain to groups at risk at birth and whose lives could be saved with effective intrapartum care.

**Fig. 2:**
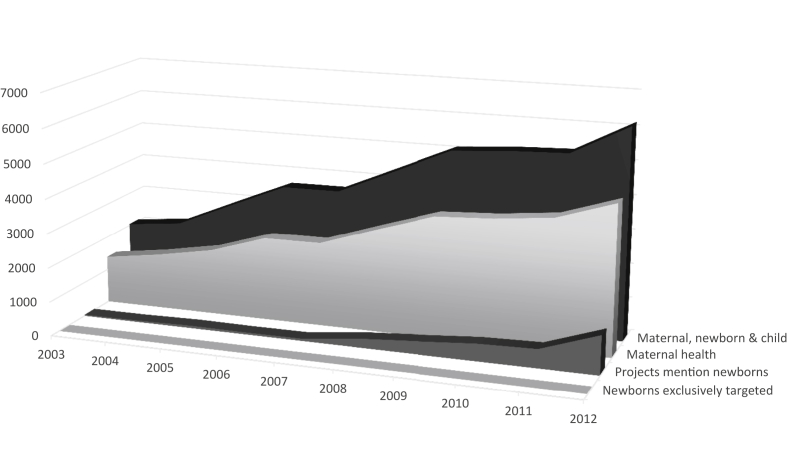
ODA for maternal, newborn and child health, 2003–2012. Note: In constant 2012 US$ (millions). Source: Arregoces et al., 2015.

This study seeks to explain how maternal and newborn survival gained status as international health and development priorities, and why they did so in an order and to a magnitude not readily predicted by existing theory. Several factors understood to facilitate issue ascendance—including certain characteristics of the issues, the existence of policy entrepreneurs and concerned actor networks, resonating issue frames and favorable international norms (Fukuda-Parr and Hulme, 2011, Keck and Sikkink, 1998, Kingdon, 1994, McInnes et al., 2012, Price, 1998, Shiffman and Smith, 2007, Shiffman, 2016a, Shiffman et al., 2016b; Snow et al., 1986)—are present in both cases. All other things equal, higher mortality and morbidity burdens among newborns and the cost-effectiveness of interventions would seem to predict that issue's earlier and higher degree of prioritization; however, the opposite has occurred. Maternal survival began to emerge as a priority some two decades in advance of newborn survival (the mid-1980s compared to the mid-2000s), and at the close of the United Nations Millennium Development Goals (MDG) era had attracted considerably more attention and resources (Fig. 2; Arregoces et al., 2015).

Issues that are unsuccessful or ‘lag’ in gaining status on organizational and political agendas are rarely examined, but their study in relationship to successful cases promises to help refine existing theory (Carpenter, 2007). We conducted replicative process-tracing case studies examining the emergence and growth of political priority for maternal and newborn survival, probing reasons for unexpected variance between the cases (Yin, 2014). We find that maternal survival's relative advantage stems from its grounding as an issue of social justice for women, which closely aligned the issue with powerful international normative forces and spurred development of a strong and diverse concerned actor network. Newborn survival's disadvantage stems from its long status as a hidden issue falling under the umbrellas of maternal and child survival but not fully adopted by either of these networks, its limited appeal as a public health issue advanced by a small and technically oriented network, and its late alignment with international normative forces.

In the sections that follow we review explanations for variation in agenda setting outcomes, drawing on theory that considers the role of ideational factors. We then present historical case studies tracing the emergence and growth of policy attention and resource allocations to maternal and newborn survival through to the launch of the Sustainable Development Goals in September 2015. In the discussion, we delineate findings and consider their implications for global health and international development priority-setting processes.

## 1 Agenda setting for global health issues

Social constructivists contend that actors are motivated not just by a logic of consequences (rational and self-interested calculations concerning the likely effects of a presumed course of action), but also a logic of appropriateness (what they perceive is right to do) (Olsen and March, 1989). They posit that principled ideas in the form of norms—shared expectations for the behavior of actors with a given identity (Katzenstein, 1996)—influence the behavior of nation-states and other international actors (Finnemore and Sikkink, 1998). Norms vary in strength. Finnemore and Sikkink (1998) elaborate a life cycle model concerning how norms advance through the international system, gaining strength as they do. In the first stage, entrepreneurs comparable to those described by Kingdon (1994) propose new standards and expectations for behavior by states and other international actors. A critical mass may accept these standards, facilitating a norm cascade across the international system. Finally, norms may become internalized—taken for granted and no longer debated. The MDG framework represents a set of strong international development norms that progressed through this life cycle, with significant implications for the policy agenda status of included issues (Fukuda-Parr and Hulme, 2011, Rushton, 2010, Smith and Rodriguez, 2016).

Alignment with strong and favorable norms—what Price (1998) terms grafting—increases an issue's chances of acquiring agenda status (Rushton, 2010, Cortell and Davis, 1996). Alignment is achieved through framing. Actors employ issue frames (ideational lenses through which problems are understood and portrayed) as political strategy, to shift understandings, attract attention and guide future action (Finnemore and Sikkink, 1998, McInnes et al., 2012, Reubi, 2012, Snow et al., 1986). Issues are more likely to garner attention when entrepreneurs and networks construct widely resonating ideational frames that convey a problem's severity, amenability to intervention, harm to at risk individuals and legal equality of opportunity (Keck and Sikkink, 1998, Shiffman and Smith, 2007, Snow et al., 1986, Stone, 1989).

Networks also seek to assemble broad coalitions to engage in collective action. Those networks that link a diverse range of actors (including advocates, scientists, donors, national and international policy makers and politicians) and have status and access are more likely to attract policy attention and resources to their issues than those that are uniform and less resourced (Hong and Page, 2004, Page, 2007, Shiffman, 2016a, Shiffman et al., 2016b). Such networks have facilitated the rise of HIV/AIDS, tuberculosis and tobacco control on the global health agenda (Gneiting and Schmitz, 2016, Kapstein and Busby, 2013, Quissell and Walt, 2016).

Carpenter (2007, 116) suggests that some issues may not be readily adopted because they overlap the concerns of multiple networks, leading advocates “to ‘pass the buck’ to experts in adjacent issue areas” and contributing to disagreements over how to frame a given issue. For instance, she asks whether ‘children born of war’ are more appropriately framed as a child protection, a support to mothers issue, or both. Other neglected health issues, such as nutrition, water and sanitation, face similar overlapping network and framing challenges.

To summarize, social constructivist theory and research suggest divergence in global priority across issues is likely attributable to differences in: (1) network strategies and strength; (2) the construction and resonance of issue frames; and (3) alignment with strong and favorable norms. Carpenter (2007) suggests the causes of issue emergence or non-emergence on advocacy network agendas deserve further investigation because these phenomena shape higher-level agenda setting processes and are under-theorized. Guided by this scholarship, we explore factors explaining differential priority for two similar global health issues.

## 2 Methodology

We used a replicative case study methodology involving process-tracing to investigate social and political processes, with the aim of uncovering causal mechanisms that affect policy outcomes (Bennett, 2010, Yin, 2014). The same research questions guided each case study since we planned to analyze the cases independently and comparatively. This study asks how policy attention and resource allocations changed for each issue over time and what factors most significantly shaped changes in agenda status. Individual case studies have been published independently and provide a foundation for this comparative analysis (Shiffman, 2016; Smith and Rodriguez, 2016). The studies were granted exempt status by Institutional Review Boards at each researcher's university.

We used multiple sources of data for purposes of triangulation and to minimize bias (Brady and Collier, 2010, Yin, 2014), including key informant interviews, published research, observation of professional meetings, and documents from donors, governments, non-governmental and other organizations. We consulted and analyzed more than 600 reports, strategies, plans, white papers, policy statements, media reports, scholarly journal articles, editorials and comments, meeting and background documents, reports to funders, press releases and public statements for their relevance to informing the research questions.

We also analyzed data from interviews with a purposeful sample of 66 key informants (42 neonatal, 24 maternal) between 2009 and 2015. We conducted semi-structured interviews guided by our research questions and analytical framework with individuals with close knowledge of issue frames, normative and funding environments, key actors and policy agenda status. We interviewed individuals representing United Nations agencies, bilateral and multilateral donors, private foundations, non-governmental organizations, research and academic institutions and professional organizations (Box 1). Interviews lasted an average of one hour and were recorded and transcribed. Data were coded and analyzed into a thematic database guided by our analytical framework; this was done by hand in the maternal case and using NVIVO9 qualitative data analysis software (QSR International, Melbourne, Australia) in the neonatal case.

We employed several strategies recommended by case study methodology experts to limit the risk of bias that accompanies research relying heavily on interviews with involved actors (Brady and Collier, 2010, Gerring, 2012, Yin, 2014). First, we triangulated data, using published and independent sources alongside interviews. We sought to independently confirm or correct accounts of events by checking published literature or reports and consulting multiple respondents. Also, we incorporated feedback on drafts of the individual case reports from five individuals familiar with the historical trajectories of the issues.

## 3 Results

Maternal survival emerged as a global health priority earlier (beginning in 1987) and has risen higher on the international health and development policy agendas than has newborn survival (which began to receive global attention only in the 2000s). We present findings for each case individually here and discuss findings from the comparison in the discussion.

### 3.1 The emergence and growth of maternal survival as a political priority

Maternal health began to attract attention as a core women's rights issue in the 1970s. Women's rights activists helped secure resolutions calling on nations to improve maternal health care at international conferences connected to the United Nations Decade for Women between 1975 and 1985. They also shaped maternal health's inclusion in the Convention on the Elimination of All Forms of Discrimination against Women, a binding treaty entered into force in 1981 (United Nations, n.d.). Activists thus framed maternal health as an issue of social justice for women, grafting the issue onto a cascading women's rights norm that advanced expectations for international action on women's health issues.

The increase in attention to maternal health in key international forums prompted United Nations agencies to begin to study and formulate a response to the global maternal mortality problem in the 1980s (WHO, 1990). The World Health Organization (1990) held its first interregional meeting on the issue in 1985, revealing that an estimated 500,000 women died annually of pregnancy-related complications. Around the same time, maternal health experts began to call attention to the disproportionate focus on children in maternal and child health programs in low-income countries—a concern captured by Allan Rosenfield and Deborah Maine's 1985 *Lancet* article titled, “Maternal mortality—a neglected tragedy. Where is the M in MCH?” The normative pressure cultivated by women's rights activists, scientific meeting and experts' criticism combined to open a window of opportunity to draw greater attention and resources to maternal survival (AbouZahr, 2001, Shiffman and Smith, 2007; Smith and Rodriguez, 2016).

Representatives of several global health and development organizations began to engage in collective action, with specific aims to reduce maternal mortality and morbidity. WHO, World Bank and the United Nations Population Fund (UNFPA) organized the first international Safe Motherhood Conference in Nairobi, Kenya, and launched the Safe Motherhood Initiative in 1987. They joined United Nations Development Programme (UNDP), International Planned Parenthood Federation and the Population Council under the rubric of the Inter-Agency Group for Safe Motherhood to formalize an advocacy network that same year. Family Care International served as secretariat. The network framed the problem as global, significant, representing the unequal status of women and amenable to change (WHO, 1990).

Following the Nairobi conference, network actors sponsored a series of regional and national conferences that more than 80 countries participated in by 1992, facilitating widespread development of national safe motherhood committees and official strategies (Otsea, 1992, Starrs, 1998, Family Care International, 2007). International organizations and agencies initiated 20 new programs aiming to improve maternal health (up from six) between 1987 and 1992 (Otsea, 1992, Family Care International, 2007). Bilateral donors (such as the United States Agency for International Development [USAID]), foundations (such as MacArthur, Ford and Gates) and university-based researchers (such as those at Columbia and Aberdeen) among others, sponsored a growing number of research initiatives, supplementing and strengthening efforts of formal network members (Otsea, 1992, UNFPA., 2004).

Network expansion, framing that closely linked the issue to a powerful women's rights norm, and efforts to bring the issue onto the agendas of influential international development forums facilitated emergence of maternal survival as a priority in the 1990s. Emergent priority is reflected in commitments of 179 International Conference on Population and Development (ICPD) Programme of Action signatories to reduce maternal mortality by half globally from 1990 levels by 2000 and a further half by 2015 (United Nations, 1995). The 1995 Fourth World Conference on Women in Beijing Platform for Action and Organization for Economic Cooperation and Development (OECD, 1996) statements make the same commitment.

At the turn of the century, United Nations Secretary-General Kofi Annan leveraged these forums and the common goals they set to promote an international poverty reduction norm represented by the United Nations Millennium Declaration and Goals. The framework's design (setting 8 specific goals) and unanimous support from heads of state lent the emergent international development norm robust agenda setting power in global and national governance arenas (Fukuda-Parr and Hulme, 2011, Shiffman and Sultana, 2013; Smith and Neupane, 2011; Smith and Rodriguez, 2016). MDG 5, to reduce maternal mortality by three quarters between 1990 and 2015, came about due to precedent—progress toward social equity for women having been established as a priority—and political feasibility. Goal 5 mirrors ICPD, Beijing and OECD commitments made just a few years earlier. These forums promoted women's reproductive health, as well, but the issue drew opposition from conservative leaders; safe motherhood advanced as a politically feasible consensus goal (Crossette, 2004, Hulme, 2009; IM24).

Maternal survival was thereby swept up in a powerful norm cascade that leaders furthered over the next decade by integrating the MDGs into organizational, national and high-level agendas and activities (Fukuda-Parr and Hulme, 2011; Smith and Rodriguez, 2016). For instance, WHO, World Bank, UNFPA and other partners provided technical and financial support for 33 countries in Africa to develop or improve national maternal health plans and integrate newborns (Box 2; de Bernis and Wolman, 2009). Norm leaders also coordinated several high-profile reports and events in 2005, including: the United Nations Millennium Project report; WHO, UNICEF, UNFPA and UNDP's flagship annual reports; and the United Nations World Summit.

As the international development norm cascaded, network actors framed maternal, newborn and child survival—and their links to Goals 4 and 5 (reduce child and maternal mortality, respectively)—as closely related issues requiring joint address. WHO presented the new framework in its 2005 *World Health Report*. Donors spurred leaders of the separate networks to align under the new Partnership for Maternal, Newborn and Child Health, forming a single secretariat housed at WHO headquarters (IM1; IM5; IM20; IM22). And, ministers and delegations from several countries supported the Delhi Declaration on Maternal, Newborn and Child Health. The integrative framing, newly aligned leadership structure and increasingly influential norm and its backers shaped subsequent high-level initiatives (Smith and Rodriguez, 2016).

Norwegian Prime Minister Jens Stoltenberg launched one of the first high-level initiatives, the *Global Campaign for the Health MDGs*. The Partnership for Maternal, Newborn and Child Health and *World Health Report 2005* were instrumental in bringing maternal health onto the agenda of what started as an effort to advance the child survival MDG (Godal, 2007, Stoltenberg, 2007) (IM5; IM19; IM20; IM24). Stoltenberg formed a Network of Global Leaders featuring UK Prime Minister Gordon Brown, Bill and Melinda Gates and the presidents of Indonesia, Mozambique and Tanzania to support the effort. Norm leaders organized several reports, strategies and events that drew attention and resources to maternal survival over the next few years, including: maternal survival series published in the prominent medical journal *The Lancet* (2006, 2007, 2013); international Women Deliver conferences (2007, 2010); Task Force on Innovative International Financing for Health Systems (2008–9); and global Consensus for Maternal, Newborn and Child Health (2009). These shaped the 2010 *Global Strategy for Women's and Children's Health*—an initiative designed to leverage the influence of the MDGs and their champions.

Committed to advancing the Goals and concerned by relatively slow progress on MDGs 4 and 5, United Nations Secretary-General Ban, 2009, Ban, 2010a) and Gates Foundation leaders developed the idea for a global strategy to spur investment and progress in late 2009 (IM5). The Partnership for Maternal, Newborn and Child Health worked alongside the Secretary-General's office to secure strong technical content, support and resource commitments (United Nations Office of the Secretary United Nations Office of the Secretary General, 2010, Ban, 2010b; IM5). Ban called on world leaders to invest in the Joint Action Plan that would become the *Global Strategy for Women's and Children's Health*. On the eve of the *Global Strategy's* launch, the estimated funding gap for maternal, newborn and child health was US$88 billion between 2011 and 2015 (Ban, 2010b). One hundred and eleven stakeholders, including governments, nongovernmental organizations, private foundations and companies had committed to support the *Global Strategy* by the time of its launch; the number of stakeholders adding commitments had tripled by August 2015 for a total of US$60 billion in financial commitments—US$22 billion new and additional funding (Partnership for Maternal, Newborn and Child Health, 2015).

International expectations, increasing national level political support and high-level leadership drew significant policy attention and resources to maternal survival through 2015. The issue's agenda status is reflected in: national strategies and policies (for instance, Ghana's MDG acceleration framework and removal of user fees for maternal health services, India's National Rural Health Mission and Tanzania's One Plans); the 2012 London Family Planning Summit; the 2014 *Every Newborn Action Plan*; and the post-2015 international development agenda. In April 2014, representatives of WHO, USAID, UNFPA, Maternal Health Task Force, Maternal and Child Health Integrated Program and 30 countries agreed on a global maternal mortality reduction goal of fewer than 70 deaths per 100,000 live births by 2030 (USAID, 2014). Advocates then leveraged their extensive network of allies to push for the new goal's inclusion in the Sustainable Development Goals—it is represented in SDG 3.1.

Increasing prioritization of maternal survival since 2000 is reflected in resource allocation trends. Analyses of development assistance for maternal health indicate funding for the issue has grown significantly since 1990 and at an increased rate in the MDG era (Arregoces et al., 2015, Institute for Health Metrics and Evaluation, 2016). Arregoces and colleagues (2015) report Official Development Assistance (ODA) for maternal and newborn health in 75 Countdown to 2015 priority countries grew from $1.4 billion in 2003 to $4.4 billion in 2012; Fig. 2 shows funding allocations to maternal and newborn health, respectively, during the period.

### 3.2 The emergence and growth of newborn survival as a political priority

Neonatal mortality was a hidden problem through the 1990s. Newborn deaths were under-reported—vital registration systems were underdeveloped and, in many societies, children were only recognized once they survived several weeks (WHO, 1996). And, the problem was commonly understood to be intractable in resource-poor settings, where high-tech hospital units and specialists were scarce (Darmstadt et al., 2014; Shiffman, 2010; Save the Children USA, 2001). Limited treatment by the 1990 World Summit for Children (which set a neonatal tetanus elimination goal) and neglect of neonates in Integrated Management of Childhood Illness, WHO and UNICEF's flagship child survival program of the 1990s, reflect limited recognition of the problem and its solutions prior to the 2000s.

A widespread assumption that newborn health needs were already being addressed by maternal and child survival initiatives and programs such as WHO's Maternal Health and Safe Motherhood Programme also contributed to issue neglect (Save the Children USA, 2001). Newborns were covered to a degree, but not to the extent needed to significantly reduce mortality (Darmstadt et al., 2005, Save the Children USA, 2001). In addition, concerns that focusing on babies would shift attention away from mothers and fears that dividing the child survival initiative into narrower concerns would weaken that cause might have prevented established maternal and child survival networks from taking the issue up more fully (Lawn et al., 2006, Shiffman and Smith, 2007; Shiffman, 2016) (IN14; IN21; IN26; IN30).

New evidence and recognition of neglect in existing programs emerged in the late-1990s, providing a foundation for public health-oriented advocacy to address the issue. WHO (1996) released the first global estimates of the problem, indicating that more than 5 million neonates died in 1995. WHO and UNICEF began to work with countries to adapt Integrated Management of Childhood Illness to address the neonatal period (Smith, 2014, Tulloch, 1999). And, a seminar at Johns Hopkins University introduced a group of individuals concerned with perinatal deaths in low-income settings to Indian physician Abhay Bang's work (Child Health Research Project, 1999). Bang and colleagues (1999) demonstrated that home-based neonatal care delivered by village women could substantially reduce mortality—high-tech hospital units and specialists were not needed to address many causes of newborn deaths.

Influenced by Bang's research, the seminar report and data on neonatal mortality rates, leaders of Save the Children USA's health and nutrition program soon proposed the idea for a global program on newborn survival to the health chief at the Bill and Melinda Gates Foundation (IN3; IN8). The Foundation granted US$50 million for a five-year program; launched in 2000, Saving Newborn Lives supported research to improve neonatal survival and promoted adoption of effective interventions with an initial focus on six countries (Bangladesh, Bolivia, Malawi, Mali, Nepal and Pakistan) and smaller programs in seven more (Save the Children USA, 2000, Save the Children USA, 2006; Shiffman, 2016; IN8). The Foundation (2014) provided a $76 million grant to support a second phase of the program from 2005 to 2011.

Saving Newborn Lives also sought to create a network of individuals and organizations working internationally on behalf of newborn survival. The program joined other major global health organizations, including Johns Hopkins University, USAID, UNICEF, the World Bank and WHO, in creating the Healthy Newborn Partnership in 2000 and served as its secretariat. The alliance aimed to raise awareness of the problem and its solutions, and to facilitate communication among concerned organizations (Lawn et al., 2006). These developments and a series of technical consultations following the Saving Newborn Lives launch helped establish an informal network of about 15 health researchers and officials in the early 2000s (IN15; IN18; IN19; IN22; IN24; IN27; IN28; IN31). Members of this small group of public health professionals would be at the center of most major newborn survival advocacy efforts through 2016, including convincing the global health organizations that employed most of them to become involved.

Convincing the major global health organizations to take up newborn survival involved establishing it as an issue that deserved attention alongside child and maternal survival. Advocates began to press for attention to M*N*CH (italics added) at UN meetings and in other public forums (IN12; IN13; IN5; IN14; IN10; IN26). For instance, an entrepreneurial actor worked with the *Lancet's* editor and other network members to develop a series on newborns (I15; see http://www.thelancet.com/series/neonatal-survival). The series helped to disseminate key public health arguments for attention to newborns, including that four million babies die in the first month of life and three-quarters of these babies can be saved with low-cost and low-tech interventions; the series also aligned the issue with international norms, arguing that MDG 4 cannot be achieved without decreasing neonatal mortality (Shiffman, 2010). In processes connected to the *Lancet* newborn survival series, WHO's 2005 *World Health Report* came to feature a chapter on newborns (alongside chapters on maternal and child health) and the Partnership for Maternal, Newborn and Child Health formed to advocate for joint address of the respective issues (this also prompted by donors) (Lawn et al., 2006).

Public health framing combined with leadership from strategic network actors and growing normative pressures (the MDGs) to increase policy and program attention to newborn survival over the next several years, though through 2010 this mainly involved health organizations as opposed to a broader set of political actors (Shiffman, 2016). For instance, following publication of the 2005 *Lancet* newborn survival series, 20 African governments approached WHO for technical advice (Lawn et al., 2006). WHO's Regional Office for Africa and donors encouraged African governments to develop newborn health strategies supporting the MDGs; 33 had done so by 2008 (Box 2; deBernis and Wolman, 2009). National and regional plans were also developed in South Asia and South America (Pan American Health Organization, 2008, Shiffman and Sultana, 2013, Smith and Neupane, 2011). Beginning in 2008, the Countdown to 2015 MDG monitoring and accountability initiative added newborn and maternal survival to its child survival mandate, the Gates Foundation began a run (2008–2014) of providing $565.3 million in grants with large neonatal components, and USAID followed ACCESS, a $75 million maternal and newborn health program covering 2004–7, with Maternal and Child Health Integrated Program, a $600 million program working in 50 countries.

Newborn survival emerged for the first time as a major item on the agendas of three inter-state institutions with broader political mandates in 2010. The Partnership for Maternal, Newborn and Child Health (2011) lobbied officials to ensure the inclusion of its issues. Donors subsequently pledged $7.3 billion for maternal, newborn and child health at the Muskoka G8 summit, the African Union made a formal declaration of support, and United Nations Secretary-General Ban-Ki Moon launched the *Global Strategy for Women's and Children's Health*. Roughly a quarter of the US$40 billion initially committed to the *Global Strategy* included newborn survival as a component (Darmstadt et al., 2014).

In 2012, the Partnership for Maternal, Newborn and Child Health (2012) worked with the Inter-Parliamentary Union to pass a resolution calling for parliaments to pursue MDGs 4 and 5. Network actors launched Born Too Soon, a global report on prematurity that generated more than 30 pledges from governments, donors, UN agencies and other organizations (IN38) (March of Dimes et al. (2012)). And, the governments of Ethiopia, India and the United States launched A Promise Renewed, an initiative in support of the *Global Strategy* that engaged national political leaders in ending preventable child deaths; 178 governments pledged to implement it and more than 30 aligned their national strategies with the initiative (UNICEF, 2015).

These developments helped lead to creation of the first global strategy on newborn survival, the *Every Newborn Action Plan*. Network actors, including those working in USAID, Saving Newborn Lives, UNICEF, WHO and the Gates Foundation, came up with the idea and secured sponsorship for the first global newborn survival conference, held in Johannesburg, South Africa in April 2013 (Shiffman, 2016) (IN34; IN36; IN38). In May 2014, 194 member states endorsed the *Every Newborn Action Plan* in a resolution at the 67th World Health Assembly; 40 commitments had been made to the plan by philanthropic foundations, UN agencies, civil society and other organizations at its launch, although a number represent prior pledges (World Health Organization, 2014). The World Health Organization and UNICEF (2015) report that 15 of 18 countries with very high neonatal mortality rates developed, were in the process of producing or enhanced existing health plans to address newborn needs since the *Every Newborn* initiative commenced.

At the close of the MDG era, global actors involved in formulating the SDGs initially focused their attention on sustaining an under-5 mortality target mirroring MDG 4 (which makes no mention of newborns) (Shiffman, 2016a, Shiffman et al., 2016b). Initiatives and advocacy by newborn survival proponents influenced the decision to add a neonatal mortality target. Particularly influential was their organization of the global conference on newborn survival in 2013, their creation of the *Every Newborn Action Plan* in 2014, their links with the UN-led Every Woman Every Child movement and advocacy within their own organizations, many of which participated in SDG development. The target included in the SDGs—‘to reduce neonatal mortality to at least as low as 12 per 1000 live births’—came from the *Every Newborn Action Plan*.

Analyses suggest ODA for newborn health grew in the MDG era (Arregoces et al., 2015, Darmstadt et al., 2014, Institute for Health Metrics and Evaluation, 2016). In one of the few studies to disentangle funding for newborns from maternal and child health, Arregoces and colleagues (2015) report on ODA for newborn health in 75 Countdown to 2015 priority countries; ODA for projects that mention newborns grew from US$33 million in 2003 to US$1.1 billion in 2012 while ODA for projects exclusively targeted to newborns grew from US$850,000 in 2003 to US$6 million in 2012. Nevertheless, newborn funding lags that for maternal and child health (Fig. 2; Arregoces et al., 2015); only about 4% of child health investments go to newborn health (Darmstadt et al., 2014). These figures reflect the relative status of the issues; newborn survival has come onto the agenda, but to a limited degree compared with maternal and child survival.

## 4 Discussion

Despite the relatively high burden of neonatal mortality globally, maternal survival emerged earlier and has risen higher on the international health and development agendas. Several factors understood to facilitate the ascendance of issues on policy agendas are present in both cases, but results vary unexpectedly. Differing conditions as the issues gained attention initially, and at the emergence of networks specifically focused on addressing them, suggest ways in which agenda setting theory might be refined.

Maternal survival first appeared on the agenda of women's rights activists, who grafted the issue onto a rapidly expanding international norm that set expectations for action to secure social justice for women around the world. This prompted several major global health and development organizations to study the problem and form a network to address it; it also brought the issue onto the international development agenda. The favorable normative environment provided the emergent network a strong platform for action as members sponsored safe motherhood conferences, facilitated development of national committees and strategies and worked to put the issue on the agendas of major international forums. International norms and maternal health advocates generated a sense of urgency that resonated beyond the health sector, securing a place for the issue on the agendas of high-level political actors. These factors set the issue up for inclusion among the MDGs at the turn of the century and for status as SDG 3.1.

By contrast, newborn survival first appeared at the margins of the maternal and child survival network agendas, drawing only some very limited programmatic attention to the issue until very recently. WHO released the first global estimates of the problem and some solutions in 1996—this stimulated no observable change in the behavior of either network toward the neonatal issue or its agenda status, however. Carpenter's (2007) buck-passing hypothesis is one possible explanation for the failure of these networks to adopt the issue more fully; our cases offer some evidence that limited adoption might alternatively be explained by views that networks are already addressing issues (e.g. within maternal and child survival programs) and perceptions of significant downsides (such as competition or dilution).

Dedicated network attention to neonatal mortality emerged only in the early 2000s. Advocates argued for attention to newborns in maternal and child survival programs, emphasizing public health arguments (high-burden problem with low-tech/community-based solutions) that resonated among health professionals and organizations. The network began to expand and policy attention to increase as efforts to graft the issue onto the child survival MDG began to pay off circa 2010, when political leaders with interests beyond the health field started to engage the issue. Newborn survival's inclusion alongside child survival in SDG 3.2 is evidence of the network's success in cultivating allies and grafting the issue onto the norm represented by the child survival MDG.

## 5 Conclusions

Comparison of the maternal and newborn survival cases offers insights to priority setting for global health issues more broadly. One implication of the study is that early attention—pre-network—to issues can lay more or less facilitative groundwork for network emergence and priority generation. Women's rights activists established maternal survival as a social justice issue, grafting the issue onto a rapidly expanding global norm; this granted the issue a degree of status on the international health and development policy agendas early on and was the base from which the maternal survival network emerged to advance its issue. By contrast, maternal and child survival networks failed to bring newborn survival more fully into their folds; as a result, the emergent newborn survival network faced the early task of establishing the issue as one that deserved agenda status alongside two issues perceived to already cover it. Hence, a second related implication of the study is that network adoption dynamics influence the emergence of political priority for issues.

A third implication is that policy attention expands with coalition building and issue frames that extend beyond health circles. Maternal survival benefited from widely resonating social justice framing and broad political coalition building. Newborn survival benefited when the issue was framed in relationship to the child survival norm and the network of concerned actors began to diversify.

Lastly, a norm layering dynamic may also influence an issue's agenda status. Successive international norms (women's rights and MDGs) bolstered maternal survival's status on the global health and development agendas. Child survival likely benefited from the same kind of layering effects—from a child rights norm pre-2000 and MDG influence after; newborn survival has benefitted little from this dynamic because it was only recently grafted onto the child survival norm in a meaningful way. The SDG era will offer the opportunity to further investigate this hypothesis. Global health networks should remain ready to adapt to changes in the normative environment and take advantage of opportunities to expand their coalitions.

## Funding

This study was supported by The Bill and Melinda Gates Foundation (Grant # OPPGH4831).

## Box 1 Affiliations of key informants.

**Maternal survival case**: American University, Averting Maternal Death and Disability at Columbia University, CARE, United Kingdom Department for International Development, Family Care International, Gynuity Health Projects, International Federation of Gynecologists and Obstetricians, the MacArthur Foundation, Maternal Health Task Force, the Norwegian Agency for Development Cooperation, Partnership for Maternal, Newborn and Child Health, Population Council, The Bill and Melinda Gates Foundation, IMMPACT at the University of Aberdeen, United Nations Population Fund, United States Agency for International Development, University of San Francisco, White Ribbon Alliance, Women Deliver, World Bank, World Health Organization.

**Newborn survival case**: Aga Khan University; All India Institute of Medical Sciences; Columbia University; Bangladesh Neonatal Forum; Family Care International; Bill and Melinda Gates Foundation; University of London Institute for Child Health; Johns Hopkins Bloomberg School of Public Health; London School of Hygiene and Tropical Medicine; Ministry of Health and Family Welfare-Government of Bangladesh; Ministry of Health and Family Welfare-Government of India; Ministry of Health-Government of Malawi; Ministry of Public Health-Government of Cameroon; Partnership for Maternal, Newborn and Child Health; Save the Children USA; Society For Education, Action and Research in Community Health; Saving Newborn Lives; UNICEF; University of Aberdeen; United States Agency for International Development; World Health Organization.

## Box 2 Africa's maternal and newborn health road maps.

Road map treatment of maternal and newborn survival varied markedly. Strategies to reduce maternal mortality were widely incorporated into national health plans in the 1990s; road maps of the mid-2000s aimed to strengthen existing maternal health plans. For newborn survival, the aim was to scale up from just a couple of interventions (e.g. tetanus elimination) to a full complement of supported strategies.
